# MeCBA预处理方案异基因造血干细胞移植挽救性治疗难治/复发急性髓系白血病的单中心回顾性研究

**DOI:** 10.3760/cma.j.cn121090-20231027-00234

**Published:** 2024-05

**Authors:** 芳芳 袁, 勇奇 王, 明会 李, 钢苹 李, 子夜 李, 瑞华 米, 青松 尹, 粤文 符, 旭东 魏

**Affiliations:** 河南省肿瘤医院（郑州大学附属肿瘤医院）血液科，郑州 450008 Department of Hematology, Henan Cancer Hospital/The Affiliated Cancer Hospital of Zhengzhou University, Zhengzhou 450008, China

## Abstract

2018年1月至2022年6月期间，30例难治/复发急性髓系白血病（R/R AML）患者在河南省肿瘤医院接受MeCBA方案（司莫司汀+克拉屈滨+白消安+阿糖胞苷）增强预处理挽救性异基因造血干细胞移植（allo-HSCT）。30例患者中男16例，女14例，中位年龄37（16～53）岁。同胞全相合供者移植3例，无关供者移植1例，单倍体移植26例。移植前中位化疗疗程数为4（3～22）个。移植后粒细胞、血小板植入中位时间分别为14（9～22）d、18（10～40）d，移植后30 d粒细胞累积植入率为100％，移植后100 d血小板累积植入率为96.7％（95％*CI* 85.4％～97.5％）。22例（73.3％）患者出现1～2级胃肠道不良反应，未出现3～4级脏器毒性。中位随访37.1个月，移植后3年总生存率为70.0％（95％*CI* 50.3％～83.1％），无事件生存率为65.3％（95％*CI* 44.8％～79.8％），累积复发率为21.2％（95％*CI* 9.2％～44.4％），非复发死亡率为16.7％（95％*CI* 7.3％～35.5％）。

## Abstract (en)

Thirty refractory relapsed acute myeloid leukemia（R/R AML）patients who received salvage allo-HSCT with MeCBA conditioning regimen from January 2018 to June 2022 at Henan Cancer Hospital were included, and their clinical data were reviewed. There were 16 males and 14 females among the 30 patients with a median age of 37（16–53）years. There were 3 sibling allograft donor transplants, 1 unrelated donor transplant, and 26 haplotype transplants. The median course of pre-transplant chemotherapy was 4（3–22）. The time of neutrophil engraftment was 14（9–22）days and 18（10–40）days for platelet. The 30-day cumulative incidence of neutrophil engraftment was 100％ and the 100-day cumulative incidence of platelet engraftment was 96.7％（95％ *CI* 85.4％–97.5％）. 22（73.3％）patients experienced grade 1–2 gastrointestinal reactions, and there was no grade 3–4 organ toxicity. With a median follow-up of 37.1 months, the overall survival（OS）rate, event-free survival（EFS）rate, cumulative recurrence rate（CIR）, and non-recurrence mortality（NRM）rate at 3 years after transplantation were 70.0％（95％ *CI* 50.3％–83.1％）, 65.3％（95％ *CI* 44.8％–79.8％）, 21.2％（95％ *CI* 9.2％–44.4％）and 16.7％（95％ *CI* 7.3％–35.5％）, respectively.

难治/复发急性髓系白血病（R/R AML）预后极差[1]–[2]，异基因造血干细胞移植（allo-HSCT）是其唯一的治愈方法[3]–[4]。接受常规预处理方案allo-HSCT治疗的R/R AML患者，移植后复发率高达80％，长期生存率不足20％[5]–[6]。克拉屈滨（Cladribine）是新一代嘌呤类似物，通过促进细胞凋亡来杀死增殖和非增殖期细胞[7]–[8]，可抑制腺苷脱氨酶而改变肿瘤细胞的表观状态[9]–[10]。克拉屈滨可增加白血病细胞对阿糖胞苷的摄取，与阿糖胞苷发挥协同抗白血病作用，从而增强对R/R AML患者的治疗效果[11]–[14]。本中心应用MeCBA预处理方案（司莫司汀+克拉屈滨+白消安+阿糖胞苷）allo-HSCT对30例未缓解（NR）R/R AML（NR-R/R AML）患者实施挽救性治疗，获得较好疗效。

## 病例与方法

1. 病例及信息收集：本研究纳入2018年1月至2022年6月在我院接受MeCBA方案预处理挽救性allo-HSCT的30例NR-R/R AML患者，对其临床资料进行回顾性分析。纳入标准：①诊断均符合2016年世界卫生组织髓系肿瘤和急性白血病分类修订标准[15]，患者被诊断为原发性AML或治疗相关AML；②≥2个疗程化疗未获得完全缓解（CR）；③复发后经过≥1个疗程挽救性联合化疗无效；④预处理之前均处于NR状态。排除标准：①骨髓增生异常综合征转化的AML；②处于急变期的慢性髓性白血病患者。细胞遗传学异常按照东部合作肿瘤组/西南肿瘤组标准[16]进行分类。以同胞供者作为首选，没有同胞供者的患者考虑无关供者或单倍体供者。本研究已于河南省肿瘤医院伦理委员会备案。

2. MeCBA预处理方案：司莫司汀250 mg/m^2^，−7 d；克拉屈滨5 mg·m^−2^·d^−1^，−6 d～−2 d；阿糖胞苷2.0 g·m^−2^·d^−1^，−6 d～−2 d；白消安3.2 mg·kg^−1^·d^−1^，−6 d～−3 d。预处理相关脏器毒性依据NCI-CTCAE.4.0进行评估。

3. GVHD预防：无关供者移植给予兔抗人胸腺细胞免疫球蛋白（r-ATG）1.5 mg·kg^−1^·d^−1^，−4 d～−1 d；单倍体移植给予r-ATG 2 mg·kg^−1^·d^−1^，−4 d～−1 d。所有患者均应用环孢素A（CsA）+霉酚酸酯（MMF）+短程甲氨蝶呤（MTX）预防GVHD。具体用量：CsA 2.5～3.0 mg·kg^−1^·d^−1^静脉滴注，−7 d开始，胃肠道功能恢复后改为口服，维持谷浓度150～250 µg/L，+40 d～+50 d后开始逐渐减量，+90 d～+120 d减停（未发生GVHD）；MMF 15 mg/kg每12 h 1次口服，−7 d～+30 d，+30 d剂量减半，+60 d停用；MTX：+1 d给予15 mg/m^2^静脉滴注，+3 d、+6 d、+11 d各予1次10 mg/m^2^。急、慢性GVHD的诊断和分级分别依据西雅图标准和NIH标准[17]–[18]。

4. 移植后地西他滨维持治疗：+50 d或骨髓造血恢复后开始给予地西他滨维持治疗（6 mg·m^−2^·d^−1^×5 d，每28天为一个疗程），维持治疗至移植后1年。

5. 移植相关并发症的预防和治疗：所有患者均入住百级层流病房，给予更昔洛韦（−7 d～−2 d）预防病毒感染，+1 d给予阿昔洛韦口服预防病毒。给予伏立康唑或泊沙康唑预防真菌。预处理过程中加强水化、碱化。+4 d开始给予皮下注射重组人粒细胞刺激因子（rh-G-CSF）及重组人血小板生成素（rh-TPO），移植期间输注的红细胞及血小板均经辐照处理。移植后定期监测巨细胞病毒（CMV）、EB病毒（EBV）拷贝数，必要时应用更昔洛韦、膦甲酸钠抗CMV治疗，或应用利妥昔单抗抗EBV治疗。移植后定期或病情需要时复查骨髓穿刺检测骨髓细胞形态学及嵌合状态、流式细胞术检测微小残留病（MRD）并个体化监测分子生物学及细胞遗传学异常。

6. 植入标准：连续3 d外周血中性粒细胞绝对计数（ANC）>0.5×10^9^/L为粒细胞植入，连续7 d血小板计数>20×10^9^/L且脱离血小板输注为血小板植入。采用DNA短串联重复序列-聚合酶链反应检测嵌合体，移植后21 d首次检测嵌合体。

7. 相关定义：总生存（OS）时间：造血干细胞回输至死亡或末次随访的时间；无事件生存（EFS）时间：造血干细胞回输至复发、死亡或末次随访的时间；复发：CR后外周血出现白血病细胞或骨髓原始细胞≥5％或髓外白血病细胞浸润。

8. 随访：通过查阅住院/门诊病历及电话随访收集患者及其供者临床资料。随访截止日期为2023年9月30日。

9. 统计学处理：应用Stata 15.0软件分析数据，采用Kaplan-Meier法绘制OS、EFS曲线，采用竞争风险模型计算累积复发率、非复发死亡率。移植后100 d内死亡的病例不纳入慢性GVHD分析。

## 结果

1. 患者基本资料：30例患者的临床特征见表1。

**表1:** 30例接受allo-HSCT挽救性治疗难治/复发急性髓系白血病患者的临床特征

特征	结果
移植年龄［岁，*M*（范围）］	37（16~53）
性别［例（%）］	
男	16（53.3）
女	14（46.7）
AML类型［例（%）］	
原发	27（90.0）
治疗相关	3（10.0）
初诊白细胞计数［例（%）］	
>100×10^9^/L	7（23.3）
≤100×10^9^/L	23（76.7）
移植前曾获缓解［例（%）］	
是	15（50.0）
否	15（50.0）
供者类型［例（%）］	
单倍体	26（86.7）
同胞全相合	3（10.0）
无关	1（3.3）
初诊遗传学分层［例（%）］	
低中危	21（70.0）
高危	9（30.0）
移植前骨髓原始细胞降幅^a^［例（%）］	
>50%	22（73.3）
≤50%	8（26.7）
髓外侵犯［例（%）］	
有	1（3.3）
无	29（96.7）
HCT-CI评分［例（%）］	
0~1分	9（30.0）
≥2分	21（70.0）
CIBMTR-DRI［例（%）］	
高危	21（70.0）
极高危	9（30.0）
移植前化疗疗程［个，*M*（范围）］	4（3~22）
诊断至移植间隔时间［例（%）］	
≤6个月	14（46.7）
>6个月	16（53.3）
供患者性别组合［例（%）］	
男供男	13（43.3）
男供女	10（33.3）
女供男	2（6.7）
女供女	5（16.7）
供患者血型［例（%）］	
相合	17（56.7）
不合	13（43.3）
造血干细胞来源［例（%）］	
外周血+脐带血	26（86.7）
外周血	4（13.3）
MNC回输量［×10^8^/kg，*M*（范围）］	10.0（3.9~19.6）
CD34^+^细胞回输量［×10^6^/kg，*M*（范围）］	5.9（3.3~10.4）
粒细胞植入时间［d，*M*（范围）］	14（9~22）
血小板植入时间［d，*M*（范围）］	18（10~40）

**注** HCT-CI：造血干细胞移植合并症指数；CIBMTR：国际血液与骨髓移植研究中心；DRI：疾病风险指数；MNC：单个核细胞。^a^难治患者与初诊比较，复发患者与复发时比较

2. 预处理相关毒性：根据NCI-CTCAE.4.0评估预处理相关器官毒性。22例（73.3％）患者出现1～2级胃肠道反应（恶心、呕吐、腹胀、腹泻），未出现3～4级脏器毒性。所有患者均存活至+28 d以后。

3. 造血重建：粒细胞、血小板中位植入时间分别为14（9～22）d、18（10～40）d，+30 d粒细胞累积植入率为100％，+100 d血小板累积植入率为96.7％（95％*CI* 85.4～97.5％）。+21 d首次检测嵌合体，30例患者均达到完全嵌合。

4. 移植疗效：所有患者在移植后28天内进行骨髓穿刺。30例患者移植后首次骨髓原始细胞均<5％，3例患者首次骨髓流式细胞术MRD阳性，其中1例患者间隔2周后再次复查为阴性，其余2例患者经减量免疫抑制剂后于移植后1.5个月MRD转阴。共有22例患者在移植后接受地西他滨维持治疗，中位维持治疗疗程数为6（4～8）个。

中位随访37.1个月，移植后3年OS率为70.0％（95％*CI* 50.3％～83.1％），EFS率为65.3％（95％*CI* 44.8％～79.8％），累积复发率为21.2％（95％*CI* 9.2％～44.4％），非复发死亡率为16.7％（95％*CI* 7.3％～35.5％）。至随访截止日期，共9例患者死亡，其中4例死于复发，非复发死亡5例（55.6％）（重度GVHD 2例，重症感染2例，死于颅内出血1例）。

5. 移植相关并发症：移植后100 d内共9例（30.0％）患者发生急性GVHD，中位发生时间为23（12～39）d。移植后100 dⅡ～Ⅳ、Ⅲ/Ⅳ度急性GVHD发生率分别为15.7％（95％*CI* 7.3％～35.5％）、6.7％（95％*CI* 1.7％～24.1％），2例Ⅲ/Ⅳ度急性GVHD患者累及肠道、肝脏、皮肤，应用糖皮质激素、抗CD25单抗、甲氨蝶呤、CsA、芦可替尼等治疗效果欠佳，最终死于急性GVHD。26例患者移植后生存超过100 d，其中4例（15.4％）发生慢性GVHD，移植后3年慢性GVHD累积发生率为23.4％（95％*CI* 10.3％～47.9％），中位发生时间为移植后12.1（5.3～15.6）个月，其中3例为局限型，1例为广泛型，在给予糖皮质激素、CsA、芦可替尼等治疗后均得到控制。

30例患者中有8例（26.7％）移植后出现CMV再激活，给予更昔洛韦、膦甲酸钠、静脉注射免疫球蛋白等治疗后均得到控制。共有6例（20.0％）患者移植后出现EBV再激活，其中3例为自限性，3例经利妥昔单抗治疗后好转。共有5例（16.7％）患者移植后发生出血性膀胱炎，均经水化、碱化、抗病毒等支持治疗好转。9例（30.0％）患者移植后合并细菌和（或）真菌感染，其中2例因重症感染死亡。4例（13.3％）患者移植后发生出血事件，3例消化道出血患者经内科治疗好转，1例颅内出血患者短时间内死亡。

## 讨论

在以往的报道中，R/R AML患者接受基于全身放射治疗（TBI）或白消安标准清髓预处理allo-HSCT后5年OS率为19％～22％[6],[19]–[20]。移植前强化预处理能最大限度地减少残余白血病细胞，从而获得更好的缓解，并使免疫重建更加有效，增强移植后移植物抗白血病（GVL）的效果，防止移植后复发，进而实现长期生存[21]–[22]。

克拉屈滨是新一代嘌呤类似物，通过多种机制杀死白血病细胞，包括诱导DNA单链断裂、抑制DNA合成以及通过抑制DNA甲基转移酶活性促进细胞凋亡[9]–[10]，杀死增殖期和非增殖期细胞。克拉屈滨还可抑制腺苷脱氨酶，改变肿瘤细胞的表观状态[7]–[8]。将克拉屈滨与白消安和低剂量TBI结合作为allo-HSCT的弱化预处理方案，对于高危髓性恶性肿瘤的老年患者表现出令人满意的结果[23]–[24]。一项前瞻性研究采用CLAG-M方案（克拉屈滨+阿糖胞苷+米托蒽醌+G-CSF）联合白消安和环磷酰胺（Bucy）强化预处理allo-HSCT治疗24例R/R AML患者，中位随访15.2个月，23例（96％）达到CR，2年OS率、无白血病生存（LFS）率分别为61.4％、59.4％[14]。另一项多中心前瞻性研究也采用类似强化预处理方案结合移植后预防性供者淋巴细胞输注治疗24例R/R-AML患者，移植后2年累积复发率为29.8％，OS率、LFS率分别为56.5％、50.5％[25]。本研究采用MeCBA强化预处理方案对30例处于NR状态的R/R AML患者进行挽救性allo-HSCT，中位随访37.1个月，移植后3年OS率、EFS率分别为70.0％、65.3％，略优于上述研究结果。

复发仍然是allo-HSCT失败的主要原因[26]，移植前处于NR状态的患者复发率较高，且复发后预后极差[27]–[28]，防止移植后复发的策略似乎更有助于改善此类患者的预后[29]。目前预防移植后复发的措施主要包括预防性供者淋巴细胞输注、靶向药物维持治疗及去甲基化药物等。对移植后复发的AML患者进行增强外显子组测序，观察到主要组织相容性复合体（MHC）Ⅱ类基因下调[30]，因此，移植后复发可能与化疗后复发的机制不同，主要基于对GVL效应耐药亚克隆的选择。此外，体外研究表明，当移植复发后的AML细胞暴露于干扰素-γ时，HLA-Ⅱ类分子的表达恢复到正常水平。这表明HLA-Ⅱ类分子的下调依赖于表观遗传学机制，可能解释了低甲基化试剂在预防和治疗移植后疾病复发中的作用[31]。研究表明，去甲基化药物通过使沉默基因的启动子区域去甲基化而增强白血病细胞表面肿瘤相关抗原的表达[32]。去甲基化药物对调节性T细胞、自然杀伤细胞具有调节作用，可能诱导HLA-Ⅰ类分子的重新表达[33]，故地西他滨可能在增强GVL效应并减少GVHD发生中起调控作用[34]。Xiao等[25]在移植后预防性输注供者淋巴细胞，2年的累积复发率为29.8％。本研究采用移植后地西他滨维持治疗预防复发，3年累积复发率为21.2％，与国内其他中心地西他滨联合重组人粒细胞集落刺激因子（rhG-CSF）应用于造血干细胞移植后维持治疗的研究[35]结果相当。

多项研究表明对R/R AML患者，单倍体造血干细胞移植具有更好的抗白血病效果[36]–[37]。一项对接受allo-HSCT的血液恶性肿瘤患者和国际骨髓移植注册中心（IBMTR）的分析显示，AML患者使用无关供者的复发风险较低[38]。有研究人员提出，单倍体移植可以在活动性疾病患者中发挥强大的移植物抗白血病（GVL）作用。本研究纳入的30例患者中27例为单倍体移植，可能是本组病例复发率较低的一个因素。

综上，本中心采用MeCBA强化预处理方案allo-HSCT挽救性治疗NR-R/R AML患者获得较好的疗效。本研究为回顾性研究且例数不多，以上结论有待于进一步验证。
